
JOURNAL INFORMATION
==============================
NLM Title Abbreviation: Wirtschaftsdienst
Journal ID: Wirtschaftsdienst
NLM Journal ID: 101086612

Wirtschaftsdienst (Hamburg, Germany : 1949)

ISSN: 0043-6275

ARTICLE INFORMATION
==============================
PMCID: PMC8800410
PMID: 35125549
DOI: 10.1007/s10273-022-3090-y
Article ID: 3090
Article version: 1
Subjects: Zeitgespräch

Kommunen zentral für Jahrzehnt der Zukunftsinvestitionen

Municipalities Central to Decade of Future Investments

Rietzler Katja Katja-Rietzler@BOECKLER.DE

Ref. Geldpolitik, Institut für Makroökonomie u. Konjunkturforschung, Hans-Böckler-Straße 39, 40476 Düsseldorf, Deutschland
Electronic publication date: 2022 Jan 29
Print publication date: 2022
Volume: 102
Issue: 1
First page: 27
Last page: 30
Copyright: © Der/die Autor:in 2022
License: Open Access: Dieser Artikel wird unter der Creative Commons Namensnennung 4.0 International Lizenz veröffentlicht (https://creativecommons.org/licenses/by/4.0/deed.de).
Open Access wird durch die ZBW — Leibniz-Informationszentrum Wirtschaft gefördert.
License URL: https://creativecommons.org/licenses/by/4.0/

Keywords: H54, H71, H72
issue-copyright-statement: © The Author(s) 2022

==============================
In the coalition agreement, the new federal government holds out the prospect of a “decade of investment for the future”. It thus recognises that considerable additional investment-related government spending is necessary to overcome the investment backlog in Germany and promote the socio-ecological transformation. Based on current studies, a total of 600 to 800 billion euros over ten year seems plausible, with a significant share accruing at the municipal level. The new government addresses important problems of municipal finances including significant regional disparities, but further measures are needed to enable all local communities to invest sufficiently.

Dr. Katja Rietzler ist Referatsleiterin für Steuer- und Finanzpolitik am Institut für Makroökonomie und Konjunkturforschung in der Hans-Böckler-Stiftung (IMK) in Düsseldorf.


Literatur

Bardt, H., S. Dullien, M. Hüther und K. Rietzler (2019), Für eine solide Finanzpolitik. Investitionen ermöglichen!, IMK Report, 152 und IW Policy Paper, 10.
Beznoska, M. und B. Kauder (2020), Schieflagen der kommunalen Finanzen: Ursachen und Lösungsansätze, IW-Policy Paper, 15.
BMWi (2018), Energieeffizienz in Kommunen. Energetisch modernisieren und Kosten sparen: Wir fördern das, Berlin.
Bremer, B., D. di Carlo und L. Wansleben (2021), The Constrained Politics of Local Public Investments under Cooperative Federalism, Max-Planck-Institut für Gesellschaftsforschung, MPIfG Discussion Paper, 21/4.
Deutscher Bundestag (2021), Gesetzentwurf der Bundesregierung. Entwurf eines Gesetzes über die Feststellung eines Zweiten Nachtrags zum Bundeshaushaltsplan für das Haushaltsjahr 2021 (Zweites Nachtragshaushaltsgesetz 2021), Bundestagsdrucksache, 20/300.
Jethon, A. und D. Schad (2021), Dem geschenkten Gaul ins Maul geschaut: Die erhöhte Bundesbeteiligung an den Kosten der Unterkunft (SGB II). Was bleibt an aufgabenbezogener („echter“) Kommunalentlastung in NRW für die Kosten der Unterkunft und der Eingliederungshilfe?, Der Gemeindehaushalt, 6.
KfW Bankengruppe (2021), KfW-Kommunalpanel 2021.
Koska, T., U. Jansen, O. Reutter, C. Schäfer-Sparenberg, M. Spitzner und A. Ulrich (2020), Praxis kommunale Verkehrswende — Ein Leitfaden, Heinrich-Böll-Stiftung, Schriften zur Ökologie, 47.
Krebs, T. und J. Steitz (2021), Öffentliche Finanzbedarfe für Klimainvestitionen im Zeitraum 2021–2030, Forum for a New Economy Working Paper, 3, https://newforum.org/wp-content/uploads/2021/09/FNE-WP03-2021.pdf (22. Dezember 2021).
SVR — Sachverständigenrat zur Beurteilung der gesamtwirtschaftlichen Entwicklung (2021), Jahresgutachten 2021/22: Transformation gestalten: Bildung, Digitalisierung und Nachhaltigkeit.
Scheller, H., K. Rietzler, C. Raffer und C. Kühl (2021), Baustelle zukunftsfähige Infrastruktur. Ansätze zum Abbau nichtmonetärer Investitionshemmnisse bei öffentlichen Infrastrukturvorhaben, WISO-Diskurs, 12.
SPD, Bündnis 90/Die Grünen, FDP (2021), Mehr Fortschritt wagen. Bündnis für Freiheit, Gerechtigkeit und Nachhaltigkeit, Koalitionsvertrag 2021–2025, https://www.spd.de/fileadmin/Dokumente/Koalitionsvertrag/Koalitionsvertrag_2021-2025.pdf (22. Dezember 2021).
Truger, A. (2018), Anhaltende Krise der Kommunalfinanzen in NRW — lokale Verantwortung für negative Globalisierungsfolgen?, in M. Junkernheinrich, S. Korioth, T. Lenk, H. Scheller und M. Woisin (Hrsg.): Jahrbuch für öffentliche Finanzen, 1–2018.
